# Dementia in People with Severe/Profound Intellectual (and Multiple) Disabilities: Practice-Based Observations of Symptoms

**DOI:** 10.1080/19315864.2022.2061092

**Published:** 2022-04-22

**Authors:** Maureen B.G. Wissing, Andrea S. Fokkens, Roos Dijkstra, Johannes S.M. Hobbelen, Annette A.J. van der Putten, Peter P. De Deyn, Aly Waninge, Alain D. Dekker

**Affiliations:** a Department of Neurology and Alzheimer Center, University of Groningen, University Medical Center Groningen, Groningen, The Netherlands; b Research Group Healthy Ageing, Allied Health Care and Nursing, Hanze University of Applied Sciences, Groningen, The Netherlands; c Department of Practice-Oriented Scientific Research (PWO), Alliade Care Group, Heerenveen, The Netherlands; d Academic Collaborative Center for PIMD, Groningen, The Netherlands; e Department of Health Sciences, Applied Health Research, University of Groningen, University Medical Center Groningen, Groningen, The Netherlands; f ‘s Heeren Loo Advisium, Amersfoort, The Netherlands; g Department of General Practice & Elderly Care Medicine, University of Groningen, University Medical Center Groningen, Groningen, The Netherlands; h Department of Inclusive and Special Needs Education, Faculty of Behavioural and Social Sciences, University of Groningen, Groningen, The Netherlands; i Laboratory of Neurochemistry and Behaviour, University of Antwerp, Wilrijk, Antwerp, Belgium; j Department of Neurology and Memory Clinic, Hospital Network Antwerp (ZNA) Middelheim and Hoge Beuken, Antwerp, Belgium; k Department of Health Psychology, University of Groningen, University Medical Center Groningen, Groningen, The Netherlands; l Royal Dutch Visio, Vries, The Netherlands

**Keywords:** dementia, intellectual disabilities, severe/profound intellectual (and multiple) disabilities, Down syndrome

## Abstract

**Introduction:**

Observable dementia symptoms are hardly studied in people with severe/profound intellectual (and multiple) disabilities (SPI(M)D). Insight in symptomatology is needed for timely signaling/diagnosis. This study aimed to identify practice-based observations of dementia symptoms in this population.

**Methods:**

Care professionals and family members were invited to complete a survey about symptoms. Quantitatively analyzed survey data were further deepened through semi-structured interviews with care professionals having vast experience in signaling/diagnosing dementia in this population. Symptoms were categorized using a symptom matrix.

**Results:**

Survey respondents and interviewees frequently observed a decline in activities of daily living (ADL) functioning and behavioral and psychological changes, like increased irritability, anxiety, apathy and decreased eating/drinking behavior. Cognitive symptoms were particularly recognized in persons with verbal communication and/or walking skills. To lesser extent motor changes and medical comorbidities were reported.

**Conclusion:**

Increased insight in dementia symptoms contributes to developing a dedicated screening instrument for dementia in people with SPI(M)D.

## Introduction

Life expectancy of people with intellectual disabilities (ID) has increased substantially over the last decades (Bittles & Glasson, 2004; Coppus, 2013; E. Evans et al., 2013). Since aging greatly increases the risk of dementia (Alzheimer’s Association, 2021), dementia is becoming increasingly prevalent among people with ID. Moreover, people with Down syndrome have a particularly high genetic risk to develop Alzheimer’s disease (AD); up to 75% will have developed dementia by age 65 (Wiseman et al., 2015). Consequently, dementia is becoming a greater challenge in ID care. Particularly, the pre-existing ID and (life-long) patterns of characteristic/typicalbehavior make it complex to recognize and diagnose dementia in people with ID (Dekker et al., 2015; Jamieson-Craig et al., 2010; Sabbagh & Edgin, 2015; Zigman et al., 2008). Furthermore, there are a variety of comorbidities which may result in dementia-like symptoms (Moriconi et al., 2015; Scott & Barrett, 2007). For example, there are similarities in symptoms between depression and dementia, and therefore depression could mistakenly be diagnosed as dementia, or vice versa (Dekker et al., 2015; Dierckx et al., 2008; Prasher, 2009).

Signaling and diagnosing dementia is particularly challenging in people with SPI(M)D, with an estimated IQ of less than 35 points (E. Evans et al., 2013; McKenzie et al., 2018). A diagnosis of dementia requires a decline in cognitive functioning interfering with performing ADL (American Psychiatric Association, 2013; McKhann et al., 2011; World Health Organization, 2018). Due to their low level of cognitive baseline functioning it is difficult to determine a decline in cognitive functioning resulting from the development of dementia (Ball et al., 2004; E. Evans et al., 2013). Moreover, they often need lifelong support to perform ADL, because they may have never developed specific skills. Never acquired skills cannot decline, and therefore cannot be indicative of dementia (Llewellyn, 2011; Sheehan et al., 2015). Furthermore, the frequent presence of multiple concurrent health problems in people with SPI(M)D make it highly complex to assess a dementia-related decline in functioning (Van Timmeren et al., 2017). Lastly, there are hardly any self-reported symptoms, because of limited verbal communication skills in people with SPI(M)D (Smiley & Cooper, 2003).

Another obstacle for (early) signaling and diagnosing dementia is the absence of validated and feasible direct neuropsychological tests and informant-based dementia screening instruments dedicated to people with SPI(M)D (Elliott-King et al., 2016; Esbensen et al., 2017; Fletcher et al., 2016; Hon et al., 1999; Keller et al., 2016; McKenzie et al., 2018). Therefore, it is hard to establish a general diagnosis of dementia, let alone that a diagnosis of a subtype of dementia (e.g., AD, dementia with Lewy bodies, vascular dementia or frontotemporal dementia) can be established (Burt et al., 1998; Day, 1985; Duggan et al., 1996; Margallo-Lana et al., 2007; Reid & Aungle, 1974). Currently, a diagnosis of dementia is based on multidisciplinary clinical assessment (by experienced clinicians) comprising observations, interviews with informants, such as family members and direct support professionals/caregivers and/or screening case notes (Day, 1985; Duggan et al., 1996; Evenhuis, 1990; Määttä et al., 2006; Margallo-Lana et al., 2007; Reid & Aungle, 1974; Sauna-Aho et al., 2018). However, information about the presentation of symptoms and course of dementia in this population is scarce (Wissing et al., 2021). Therefore, dementia symptoms may not be recognized or may mistakenly be attributed to the ID, resulting in a (too) late diagnosis or no diagnosis at all (Cleary & Doody, 2017). Nevertheless, it is essential to diagnose dementia in people with SPI(M)D to be able to timely respond to the persons’ changing wishes and needs by making informed choices (Dekker, Wissing et al., 2021; Janicki, 2011).

Early signaling and diagnosing dementia in people with SPI(M)D requires a proper understanding of the presentation of dementia symptoms in this population. Recently, we obtained a first inventory of observable symptoms from the scarce literature (Wissing et al., 2021) and focus groups (Dekker, Wissing et al., 2021). This study aimed to further identify and deepen observable dementia symptoms in people with SPI(M)D through a survey and semi-structured interviews.

## Methods

### Study Consortium

This study is part of the research project ‘Practice-based questions about dementia in people with SPI(M)D” (Dekker, Wissing et al., 2021; Wissing et al., 2021), a collaborative effort of Hanze University of Applied Sciences, University of Groningen and University Medical Center Groningen (UMCG) with four care institutions throughout The Netherlands (Ipse de Bruggen’s Heeren Loo, Alliade Care Group and Royal Dutch Visio). These care institutions are representative for the Dutch ID care sector given the high number of people with SPI(M)D for whom they provide diagnostic work-up, treatments and deliver care.

### Study Design

A mixed methods design was adopted comprising a survey and semi-structured interviews. Firstly, a survey was developed to identify practice-based observations of dementia symptoms in people with SPI(M)D. Secondly, interviews with care professionals were conducted to collect richer and more in-depth perspectives on symptoms covered in the survey. The Good Reporting of A Mixed Methods Study (GRAMMS; O’Cathain et al., 2008) and Consolidated Criteria for Reporting Qualitative Research (COREQ; Tong et al., 2018) were used as guidance for reporting this study.

### Ethics and Consent

The Medical Ethical Committee of the UMCG decided that the Dutch Medical Research Human Subjects Act did not apply to this study (METc 2019/198). The study was registered in the UMCG Research Register (no. 201900193) and conducted in accordance with the UMCG Research Code and the EU General Data Protection Regulation. Survey respondents provided consent for analyzing their responses by answering a consent question before the start of the survey. Interviewees provided written informed consent for audiotaping and analyzing the interview.

### Survey

#### Respondents

Care professionals and family members of people with SPI(M)D and (suspected) dementia (established according to clinical judgment and (medical) records) were invited to participate in an online Dutch survey. The project team, consisting of representatives from consortium partners, identified eligible care professionals and family members within the four participating care institutions, partly through snowball sampling. Eligible respondents were purposefully selected based on the criterion that they had relevant experience/had a relative with SPI(M)D and (suspected) dementia, and thus were able to provide information about observable dementia symptoms, i.e., purposive sampling (Palinkas et al., 2015). Consequently, respondents were excluded if they only had experience with/their relative had mild/moderate ID or when they had no experience with (suspected) dementia in those with SPI(M)D. The project team emailed the survey link to eligible respondents. A reminder was sent two weeks after initial invitation. Moreover, the survey link was disseminated via websites and newsletters of the research project and consortium partners. Five family members received a paper version of the survey due to limited computer accessibility/skills. Responses on paper were digitalized after completion.

#### Data Collection

To construct the survey, we followed the steps described by Passmore et al. (2002). The first part consisted of two closed-ended questions to check whether respondents met inclusion criteria, i.e., having relevant experience with/having a relative with SPI(M)D and (suspected) dementia. The survey ended if respondents did not meet inclusion criteria. The second part gathered demographic data about age, sex, highest level of education and relationship to people with SPI(M)D. The third part included items evaluating the observation of dementia symptoms, subdivided into four symptom domains based on diagnostic dementia criteria (American Psychiatric Association, 2013; McKhann et al., 2011; World Health Organization, 2018) and literature (Dekker, Ulgiati et al., 2021; Ries, 2018; Strydom et al., 2010). The fourth part contained an open-ended question, in which respondents were asked whether they had observed changes not addressed in the survey.

Within part three, the first domain focused on cognitive functioning. In the general population AD is the most common cause of dementia, accounting for 60–80% of all diagnosis (Alzheimer’s Association, 2021). Moreover, people with Down syndrome have an extremely high genetic risk to develop dementia due to AD (Lott & Head, 2019; Wiseman et al., 2015). Therefore, items within this domain consisted of cognitive functions affected by AD: memory, planning, problem solving, orientation in time, orientation in place, understanding visual images/spatial relationships, language skills, losing objects and judgment (Alzheimer’s Association, 2021). These items were complemented by cognitive changes addressed in the focus group study of Dekker, Wissing et al. (2021): person recognition, object recognition, preference for (favorite) objects, responsiveness and awareness of proper order. Moreover, one item within this domain focused on ADL functioning (Alzheimer’s Association, 2021). Given that dementia is characterized by a decline of cognitive and ADL functioning (American Psychiatric Association, 2013; McKhann et al., 2011; World Health Organization, 2018), response options were defined as: decrease (increase for losing objects, given that people with dementia may lose objects more frequently), unaltered, never shown or unknown.

The second domain contained behavioral and psychological items according to the sections described in the Behavioral and Psychological Symptoms of Dementia in Down Syndrome scale version II (BPSD-DS II) (Dekker, Ulgiati et al., 2021). In the BPSD-DS II, restless and stereotypic behavior constitutes one section. To ensure that the survey addressed one aspect at a time, restless and stereotypic behavior were addressed as two separate items. Although most individuals with dementia display an increased frequency of behavioral changes, decreased frequency are also observed (Dekker, Ulgiati et al., 2021). Therefore, response options were defined as: increase, decrease, unaltered, never shown or unknown.

The third domain comprised motor items: walking, balance, fall frequency, movement speed (Ries, 2018), stiffness, muscle strength, cramps, wheelchair use and choking (Dekker, Wissing et al., 2021). The fourth domain focused on medical comorbidities: epilepsy, weight and incontinence (Strydom et al., 2010). Depending on the item, response options were defined as: increase, decrease, unaltered, never shown or unknown. Moreover, each domain was followed by a comment field.

Subsequently, two survey versions were created: one for family members (answering about an individual case) and one for care professionals (answering about multiple cases). Given that the observation of changes can vary for different persons, two additional response options, 1) decrease for some persons, increase for others 2) unaltered for some persons, never shown for others, were added in the version for care professionals. The online survey versions were constructed in REDCap (Harris et al., 2009), hosted within the secured network of the UMCG.

During survey construction, project team members reviewed structure and content, leading to optimizing structure, removing redundant questions, rephrasing items. Subsequently, draft versions were pilot tested with two family members and six care professionals. Based on this pilot, the expected time needed to fill out the survey was between 10 and 15 minutes. Moreover, analysis of the pilot survey findings resulted in expanding survey introduction, refining texts (incl. shortening response options), reordering questions and changing visual presentation. The pilot survey respondents did not fill out the final survey. Final survey versions were launched in August 2020. Data collection lasted three months.

#### Data Analysis

Responses were exported to SPSS Statistics version 27 (IBM Corp), surveys which were not completed were excluded. Standard descriptive statistics and stacked bar graphs were used to present results. From left to right in the stacked bar graphs, changes per domain were depicted from most frequently to least frequently reported. Additionally, responses to open-ended fields/question were analyzed by coding symptoms as described in the interview data-analysis section.

### Interviews

#### Participants

The project team purposefully selected 28 eligible care professionals (not necessarily persons who had filled out the survey) having vast experience in signaling/diagnosing dementia in people with SPI(M)D. They were particularly knowledgeable about and experienced in dementia in people with SPI(M)D and could thus provide a richer and more in-depth perspective. Eligible care professionals were consecutively invited until data saturation was reached, which was defined as the moment no new dementia symptoms were mentioned. Furthermore, to ensure that the interviewees reflected the multidisciplinary composition of professionals in daily practice, we selected interviewees based on their profession. Until data saturation was reached, 19 eligible participants had received an invitation by e-mail. Two persons did not respond, and three persons were unable to attend because of scheduling issues. Two eligible participants suggested to include their direct colleagues (same profession, same care institution) who could provide a wealth of information as well (snowball sampling). Consequently, two interviews were held, in which two participants were simultaneously interviewed. Also, these two interviews were considered in the process of determining data saturation.

#### Data Collection

Semi-structured interviews were conducted via Microsoft Teams by one author (ASF) and lasted 45 to 75 minutes. An interview protocol was developed in advance, in consultation with project team members, and based on the guidelines by Boyce and Neale (2006). The interviewer followed instructions (protocol) which entailed a series of steps. Each interview started with welcoming interviewee(s), introducing the topic, checking if interviewee(s) had signed informed consent forms, asking permission for audiotaping, explaining procedure and confidentiality. Furthermore, interviewees were asked to provide demographic information: age, sex, highest level of education and working experience with SPI(M)D and (suspected) dementia. Subsequently, the interviewer addressed symptom domains covered in the survey by asking open-ended questions. The first question was “On the basis of which symptoms do you conclude that someone with SPI(M)D has dementia?” Next, the interviewer asked follow-up questions (protocol) or could ask additional questions about relevant brought up symptoms. Each interview ended with summarizing discussed themes, asking if they would like to share anything else and thanking the interviewee(s). After the first interview, the protocol was refined, i.e., rephrasing questions.

Interviews were recorded with a Philips audio recorder (DVT6510). To evaluate whether saturation was achieved, another researcher (MBGW) – not present during the interview – listened after each interview to the recording and summarized dementia symptoms. Data saturation was discussed with the project team members. Audiotapes were transcribed in Dutch (clean transcription) by the University Translation and Correction Service of the University of Groningen Language Center. Fillers, hesitations and slips of the tongue were left out.

#### Data Analysis

Transcripts of all 14 interviews were analyzed using a qualitative method of content analysis combining aspects of deductive and inductive content analysis (Elo & Kyngäs, 2008). Firstly, one researcher (MBGW) read all transcripts to familiarize yourself with the data. Secondly, this researcher openly coded symptoms within the transcripts in ATLAS.ti version 8 (Scientific Software Development GmbH). A second researcher(ASF) coded selected symptom text fragments of six interviews (in total 395 symptoms) by using the codes generated by the other researcher (MBGW). The number of concordant coded symptoms was 296. Intercoder percent agreement, i.e., number of concordant coded symptoms/total number of coded symptoms x 100 (Gisev et al., 2013), was 74.9%.

To structure the broad range of symptoms, a categorization matrix (Elo & Kyngäs, 2008), here called symptom matrix was designed, similar to Dekker et al. (2021). The symptom matrix rows were deductively designed in line with the symptom domains and items addressed in the survey. To further improve interpretation, the symptom matrix columns were thereafter inductively designed. Project team members discussed and refined categorization and (sub)thematization until reaching consensus. To improve trustworthiness, illustrative quotes were selected to support results (Elo & Kyngäs, 2008). One researcher (MBGW) translated selected Dutch quotes to English, which were where possible shortened (e.g., by leaving out unnecessary colloquial words). Project team members checked whether translations were accurate and if intentional meanings were maintained.

## Results

### Survey

**Figure 1.**
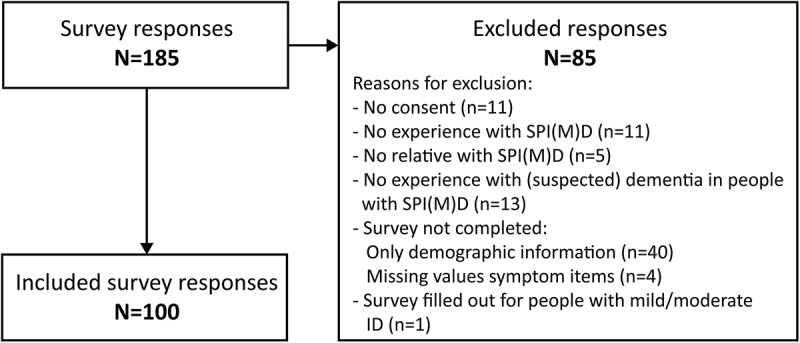
Schematic overview of included and excluded survey respondents.

In total, 185 respondents started filling out the survey, of whom 85 were excluded for various reasons (Figure 1), primarily surveys which were not completed. Of the total 85 excluded responses, 61% were from care professionals and the remaining 33% were from family members. Data of 100 respondents, i.e., 87 care professionals and 13 family members (Table 1) were eligible for analysis.

**Table 1.** Characteristics of respondents.

Characteristics	All respondents N = 100	Care professionals N = 87	Family members N = 13
Age (years [median (IQR), min-max])	46 (22), 21–83	42 (19), 21–68	63 (20), 44–83
Sex (% female)	91	94	69
Level of education: primary school, high school, mbo, hbo, wo (%)	2, 2, 37, 39, 20	0, 0, 39, 38, 23	15, 15, 23, 46, 0
Care institution: Ipse de Bruggen, ’s Heeren Loo, Alliade, Visio, other (%)		38, 21, 24, 9, 8	N/A
Role: physician, nurse specialist, DSP, psychologist, psychologic assistant, occupational therapist, speech therapist, physiotherapist, dietician (%)		6, 6, 51, 16, 2, 5, 8, 6, 1	N/A
Experience working with SPI(M)D (years [median (IQR), min-max])		15 (13), 0–44	N/A
Working with SPI(M)D: D, W, M, other (%)		39, 43, 13, 6	N/A
Experience working with SPI(M)D + (suspected) dementia (years [median (IQR), min-max])		10 (12), 0–33	N/A
Working with SPI(M)D + (suspected) dementia: D, W, M, other (%)		35, 38, 15, 13	N/A
Family relationship: parent, sibling, no family member but legal representative (%)		N/A	8, 77, 15
Years knowing relative ([median (IQR), min-max])		N/A	57 (13), 1–67
Frequency of visits (% W, M, Q)		N/A	31, 62, 8
**Characteristics of relative with SPI(M)D + (suspected) dementia**			
- Age (years [median (IQR), min-max])		N/A	60 (9), 47–73
- Level of intellectual disability: severe, profound, not determined but probably severe/profound (%)		N/A	62, 31, 8
- Presence of Down syndrome (%)		N/A	54
- Presence of multiple disabilities (%)		N/A	62
- Living situation: care institution, at home (%)		N/A	92, 8

Percentages (rounded off to the nearest whole number without decimals) are calculated based on the total number of respondents per group (column). The group of psychologists is composed of behavioral therapists who studied psychology or special needs education (in Dutch: orthopedagogiek). Abbreviations: D, daily; DSP, direct support professional/caregiver; hbo, higher vocational education; M, monthly; mbo, intermediate vocational education; N/A, not applicable; Q, quarterly; SPI(M)D, severe/profound intellectual (and multiple) disabilities; W, weekly; wo, higher education.

#### Cognitive and ADL Changes

**Figure 2.**
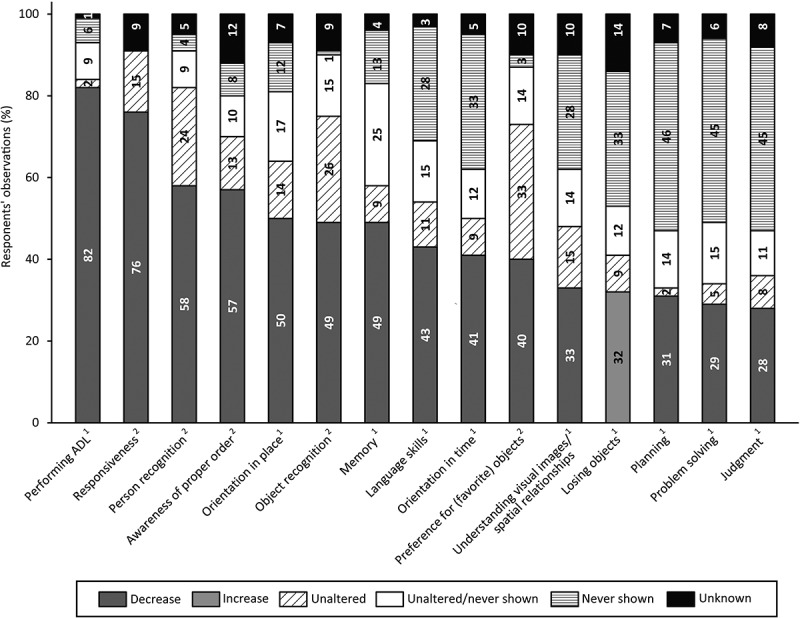
Respondents’ observations of cognitive and activities of daily living (ADL) changes in people with SPI(M)D since the onset of (suspected) dementia. Per item, the proportion (%) of decrease, unaltered, unaltered/never shown (i.e., unaltered for some persons, never shown for others), never shown and unknown are presented within each bar. From left to right, items are ordered from highest to lowest percentage of respondents observing a decrease (increase for losing objects) since (suspected) dementia. References: 1, (Alzheimer’s Association, 2021); 2, (Dekker, Wissing et al., 2021).

Figure 2 shows the respondents’ observations of cognitive and ADL changes in people with SPI(M)D since the onset of (suspected) dementia. The survey revealed that the most frequently observed dementia symptom in this population was a decline in ADL functioning (82%). Among cognitive items, most respondents (76%) indicated a decrease in responsiveness since (suspected) dementia. Less frequently changes inplanning (31%), problem solving (29%) and judgment (28%) were observed. For these items, respondents often reported that individuals had never shown these cognitive functions. Additionally, in the open text field respondents indicated that since the onset of (suspected) dementia they had observed changes in sensory sensitivities (n = 3) and a decreased ability to concentrate (n = 1).

#### Behavioral and Psychological Changes

**Figure 3.**
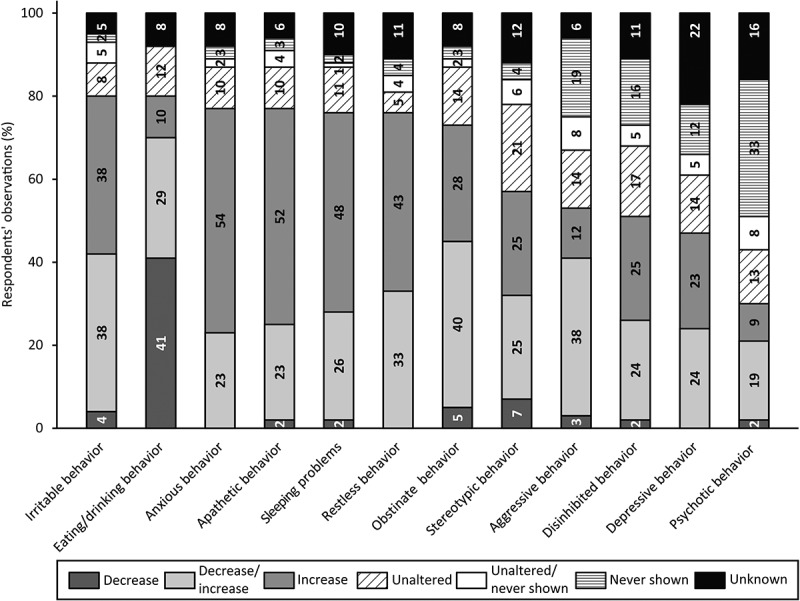
Respondents’ observations of behavioral and psychological changes in people with SPI(M)D since the onset of (suspected) dementia. Per item, the proportion (%) of decrease, decrease/increase (i.e., decrease for some persons, increase for others), increase, unaltered, unaltered/never shown (i.e., unaltered for some persons, never shown for others), never shown and unknown are presented within each bar. From left to right, changes are depicted from most frequently reported (either a decrease, an increase or a combination of both) to least frequently reported. Behavioral and psychological categories are provided in accordance with the sections of the BPSD-DS II (Dekker, Ulgiati et al., 2021).

Figure 3 provides an overview of the percentage of respondents reporting behavioral and psychological changes. Evidently, changes in irritable behavior (80%) and eating/drinking behavior (80%) were most frequently reported. For all items within this domain an increase as well as a decrease in behavior relative to the pre-existing life-long characteristic behavior was observed since the onset of (suspected) dementia. For instance, concerning irritable behavior, 38% of the respondents observed an increase, 4% a decrease and another 38% had observed a decrease for some persons and an increase for others. Moreover, individuals ate and drank less/slower according to 41%, more/faster according to 29% and variable according to 10%. Furthermore, respondents frequently highlighted changes in anxious behavior (77%), apathetic behavior (77%), sleeping problems (76%), restless behavior (76%) and obstinate behavior (73%). Changes in depressive (47%) and psychotic behavior (30%) were less commonly observed. Often respondents had never observed this behavior (12% and 33%, respectively) or they did not know whether behavior had changed (22% and 16%, respectively). Additionally, respondents reported changes in compulsive behavior, which was not addressed in the survey. One had observed an increase in compulsive behavior, whereas two others had observed a decrease relative compared to the pre-existing life-long characteristic behavior.

#### Motor Changes

**Figure 4.**
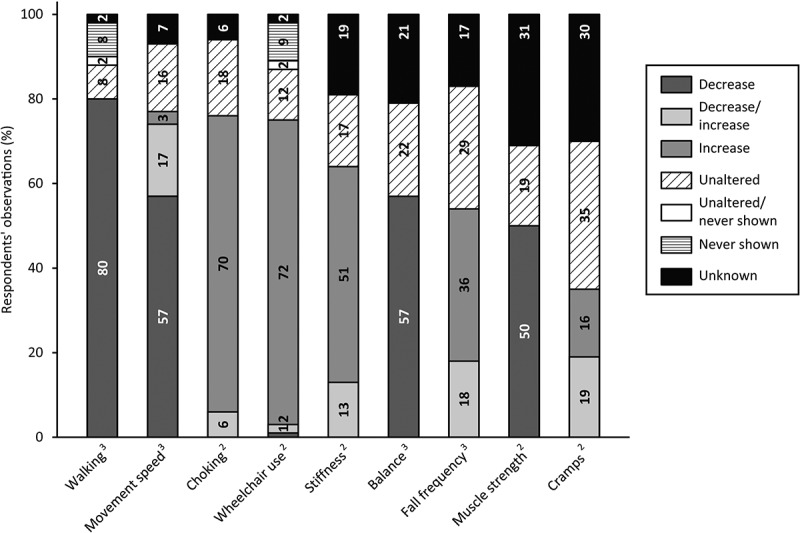
Respondents’ observations of motor changes in people with SPI(M)D since the onset of (suspected) dementia. Per item, the proportions (%) of decrease, decrease/increase (i.e., decrease for some persons, increase for others), increase, unaltered, unaltered/never shown (i.e., unaltered for some persons, never shown for others), never shown and unknown are presented within each bar. From left to right, motor changes are presented from most frequently reported (either a decrease, an increase or a combination of both) to least frequently reported. References: 2, (Dekker, Wissing et al., 2021); 3, (Ries, 2018).

Figure 4 visualizes the responses about motor changes. The majority of respondents (80%) noticed that, since the onset of (suspected) dementia, walking skills had declined. Moreover, the wheelchair was more frequently used (72%), choking was more common (70%) and movements were slower (57%) since the onset of (suspected) dementia. Changes in muscle strength and cramps were less common, often it either remained stable (19% and 35%, respectively) or unknown (31% and 30%, respectively). Additionally, respondents described motor symptoms not addressed in the survey: decreased body awareness (n = 3), decreased motor skills (n = 2), increased tremor (n = 1), sitting/laying more in fetal position (n = 1).

#### Medical Comorbidities

**Figure 5.**
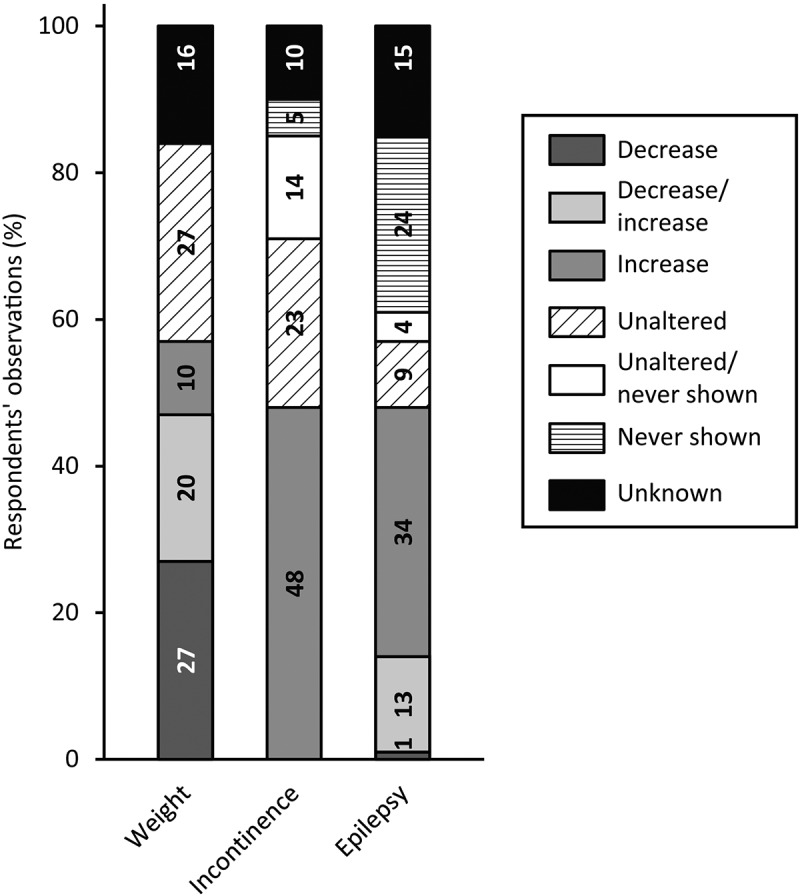
Respondents’ observations of changes in medical comorbidities in people with SPI(M)D since the onset of (suspected) dementia. Per item, the proportion (%) of decrease, decrease/increase (i.e., decrease for some persons, increase for others), increase, unaltered, unaltered/never shown (i.e., unaltered for some persons, never shown for others), never shown and unknown are shown within each bar. From left to right, motor changes are presented from most frequently reported (either a decrease, an increase or a combination of both) to least frequently reported. Reference: (Strydom et al., 2010).

Figure 5 visualizes respondents’ observations of medical comorbidities in people with SPI(M)D and (suspected) dementia. Respondents observed weight changes (57%), mainly weight loss (27%), increased incontinence (48%) and increased frequency and severity of epileptic seizures (34%). In the open text fields, three medical comorbidities not included in the survey were addressed: decreased taste sensation (n = 2), increased pain (n = 1) and becoming bedridden (n = 1).

### Interviews

Based on a first analysis, we concluded that observable dementia symptoms mentioned during interview 12, 13 and 14 were consistent with earlier interviews and thus saturation had been reached. Hence, a total of 14 interviews were conducted with 16 care professionals (Table 2).

**Table 2.** Characteristics of interviewees.

Characteristics	Care professionals N = 16
Age (years [median (IQR), min-max])	50 (21), 29–64
Sex (% female)	88
Level of education: mbo, hbo, wo (%)	13, 38, 50
Care institution: Ipse de Bruggen, ’s Heeren Loo, Alliade, Visio, other (%)	25, 19, 19, 31, 6
Role: physician, nurse specialist, DSP, psychologist, occupational therapist, speech therapist, physiotherapist (%)	19, 6, 19, 25, 13, 13, 6
Experience working with SPI(M)D (years [median (IQR), min-max])	22 (16), 5–33
Experience working with SPI(M)D + (suspected) dementia (number of people [median (IQR), min-max])	127 (186), 2–500

Percentages (rounded off to the nearest whole number without decimals) are calculated based on the total number of interviewees. The group of psychologists is composed of behavioral therapists who studied psychology or special needs education (in Dutch: orthopedagogiek). Abbreviations: DSP, direct support professional/caregiver; hbo, higher vocational education; mbo, intermediate vocational education; SPI(M)D, severe/profound intellectual (and multiple) disabilities; wo, higher education.

Reported symptoms were coded and subsequently categorized using a symptom matrix (Table 3). Inductive content analysis revealed that symptoms were generally observed in relation to having verbal communication or walking skills, which is in accordance with a survey respondent stating in open text field that the observation of dementia symptoms in persons with SPI(M)D depended on whether at baseline individuals were verbal or could walk. In addition, a category called “category-independent” was created for symptoms not affected by the presence or absence of verbal communication or walking skills

Psychologist C.: “Many persons within the SPI(M)D group are dependent on a wheelchair, they cannot walk and/or cannot talk. Then it is far more difficult to examine (…).”

**Table 3.** Symptom matrix structuring dementia symptoms in people with SPI(M)D observed by interviewees.

			Verbal communication skills	Walking skills	Category-independent
Cognitive changes^1,2,3^	Memory	P	↓ remembering names, ↑ saying incorrect things, ↑repeatedly asking something	↓ remembering: how to walk/dress up/set table, that you went to toilet, were to hang coat, that you were asked to sit on chair.	↓ understanding: what is (about to) happen, expectations, activities, changes, jokes, communication (verbal, augmentative and alternative). ↓ remembering: new information, that you were going to eat/drink, how to use cutlery, how to chew/swallow/brush teeth, what to do with food in mouth, that dining chairs belong to table.
		A			
	Orientation in time	P	Saying “good morning” in afternoon	Put on pajamas in morning, regular clothes in evening	
		A			
	Orientation in place	P		↓ remembering: where you/rooms are, direction/destination, new routes. ↑ getting lost, ↑ suddenly standing still in rooms	
		A		↑ stretching arms when passing doorways, ↑ agitation when wheelchair is turned	
	Understanding visual images/ spatial relationships	P		↑ bumping into things, difficulty with floor transitions	
		A		↑ bumping into things with wheelchair	
	Language skills	P	↓ talking, ↓ speech intelligibility, ↓ number of words used, ↓ expression with words, ↓ language comprehension, stop talking mid-sentence, speaking confusedly		
		A	↓ frequency of producing sounds		
	Losing objects	P		↓ remembering where you put toys	
		A			
	Person recognition	P			↓ recognizing DSP/family members
		A			
	Object recognition	P		↓ recognizing walking lines on floor	↓ recognizing food/cutlery/table/chair/doll
		A			
	Sound recognition	P			↓ recognizing sounds/songs
		A			
	Preference for (favorite) objects	P		Not going to preferred seat	Liking things that were previously disliked, no longer touching cup/toys that someone used to hold
		A			
	Responsiveness	P			No response while previously full of expectations, ↓ quickness of response, ↓ contact, ↓ alertness, ↑ alertness, ↓ keeping up with pace, ↓ reacting on the environment, ↑ staring ahead, ↑ not making eye contact, ↑ not responding to songs/activities
		A			
	Awareness of proper order	P		↓ understanding sequence of showering before dressing up, ↑ dependency on structure, put underwear over pants, start activities at wrong moment	↓ remembering: daily routines, consecutive steps. Shift daily routines, skip steps, ↓ understanding daily activity icons
		A			
	Sensory sensitivities	P			Sensory overload, negative reactions to stimuli, cannot bear sounds/songs/light, ↓ toleration of other residents, perceiving being touched as unpleasant, clothes feel uncomfortable, ↑ seeking proprioceptive input
		A			
	Concentration	P			↓ concentration
		A			
ADL changes^1,2,3^	ADL functioning	P		↓ ability to: (un)dress, put on socks/coat, go to toilet, stair climbing.	↓ ability to: eat/drink, take food from spoon, open mouth, use cutlery/cup, washing vegetables, fold laundry, pick up puzzle pieces.
		A		↓ ability to propel wheelchair	
Behavioral and psychological changes^4,5^	Anxious behavior	P		↑ hesitant to walk, sliding across floor	↑ screaming, ↑ crying, ↑ whining, ↑ nervous, ↑ tension, ↑ panic, ↑ stress, ↑ freezing, inability to relax, anxious facial expression, fear in someone’s eyes, eyes wide open, forehead sweating, ↑ feeling unsafe, ↑ fear of being alone/hoist/going outside/hard noises/at night/ when eating, ↑ frightened when being touched/ball is thrown, clinging to table, avoiding things, ↑ contact seeking, ↑ seeking security, hesitant to let DSP go
		A		↓ anxiety for hoist	
	Sleeping problems	P		↑ wandering at night, crawl out of bed	Day-night rhythm disturbance, ↑ insomnia, ↑ prowling about/restlessness/waking up/screaming at night, ↑ difficulty getting up, ↑ sleeping during day, ↑ being tired during day, falling asleep earlier
		A			
	Irritable behavior	P			↑ touchy, ↑ irritability, ↑ frustration, ↑ anger, ↑ yelling, ↑ screaming during ADL, ↑ sounds of discomfort
		A			
	Obstinate behavior	P		↓ willingness to walk	↑ resistance against eating/dressing up/showering/ activities. ↑ being uncooperative, ↑ being self-willed, no longer accepting aids, ↑ turning head away
		A			
	Restless/stereotypic behavior	P	↑ repeating words/questions, excessive talking	↑ walking, ↑ wandering	↑ restlessness, ↑ compulsive acts, ↑ stereotypical acts, ↑ picking behavior, ↑ fecal smearing, ↑ rituals preventing to sleep
		A			
	Aggressive behavior	P		↑ slam doors	↑ verbal/physical aggression against self and/or others, ↑ biting/beating, ↑ throwing objects
		A			
	Apathetic behavior	P		↓ motivation to walk, not getting of sofa	↑ withdrawn, ↑ being passive, ↑ laziness, ↓ initiative, ↓ motivation, ↓ jovial, ↓ enjoying food/music, ↓ interest in activities they used to like, ↓ focus on eating, ↓ (eye) contact, ↓ noticing things, ↑ staring ahead, ↑ letting go daily structure
		A		↑ sitting still in wheelchair	
	Depressive behavior	P			↓ emotion regulation, ↓ smiling, ↑ crying, ↑ discourages, ↑ mood changes, very unhappy, hollow eyes, head down
		A			
	Psychotic behavior	P	↑ mentioning things that are not there		↑ suddenly looking at something/noticing things
		A			
	Eating/drinkingbehavior	P			↓ appetite/eating/drinking, ↓ preference favorite food, eating slowly
		A			
Motor changes^5^	Motor skills	P		↓ walking distance/speed, gait changes, ↓ lower limb coordination, ↓ standing up, ↑ bottom shuffle, ↑ wheelchair use	↓ movement speed, ↑ stiffness, ↑ clumsiness, ↓ muscle strength, ↑ muscle tension, ↑ cramps, ↑ overstretching muscles, ↑ fetal sleep position, ↑ sitting cross-legged
		A			
	Balance	P		↑ gait clumsiness/unsteadiness, ↑ insecure walking, ↑ tripping, ↑ falling, ↓ body awareness	
		A		↓ maintaining body posture	
	Chewing/ swallowing	P			↓ chewing, ↓ swallowing, ↑ unsafe swallowing, ↑ swallowing without chewing,↑ choking
		A			
Medicalcomorbidities^6^		P		↑ incontinence, ↑ weight, ↓ bowel movements, ↑ bedridden	↑ epilepsy, ↓ weight
		A			

Dementia symptoms reported by interviewees were categorized based on symptom domains and items addressed in the survey (rows) and verbal communication/walking skills at baseline, i.e., highest level of functioning before dementia-related decline occurred (columns). Legend: ↓ = decrease compared to baseline level of functioning, ↑ = increase compared to baseline level of functioning. Abbreviations: ADL, activities of daily living; A, skill/behavior absent at baseline; DSP, direct support professional/caregiver; P, skill/behavior present at baseline. References: 1, (American Psychiatric Association, 2013); 2, (McKhann et al., 2011); 3, (World Health Organization, 2018); 4, (Dekker, Ulgiati et al., 2021); 5, (Ries, 2018); 6, (Strydom et al., 2010).

Table 3 shows that interviewees observed cognitive changes particularly when individuals had verbal communication or walking skills at baseline, whereas behavioral and psychological changes were mostly observed irrespective of such skills. Motor changes were particularly observed when persons were able to walk at baseline. Furthermore, changes in ADL functioning and medical comorbidities were observed in people with and people without walking skills at baseline. Results within each domain are described below in more detail, supported by quotes to clarify interviewees’ observation of symptoms.

#### Cognitive Changes

Interviewees stated that observing cognitive symptoms in people with SPI(M)D is very complex. Nevertheless, cognitive symptoms like deterioration in language skills, memory loss and disorientation in time were (mainly) recognized when individuals had verbal communication skills at baseline.

Physician T.: “Language is something that is very obvious. People are going to use fewer words and eventually stop talking. If someone has the ability to speak, then loss of speech could certainly be a signal.”

Disorientation in place, losing objects and trouble understanding visual images/spatial relationships were particularly observed people with walking skills.

Speech therapist M.: “Walking into the wrong direction or suddenly going to the toilet, but walking into the laundry room instead, that are signals when someone is able to walk. (…) That is not observable when someone is dependent on a wheelchair.”

Cognitive symptoms like reduced responsiveness, declined person recognition and increased sensory sensitivities were observed regardless of having verbal communication or walking skills. This also applied to reduced sound recognition, which was a symptom not addressed in the survey.

#### ADL Changes

Particularly, a decline in eating/drinking skills was observed in persons with SPI(M)D and (suspected) dementia. A decline in dressing, toilet use and stair climbing were only noticed in individuals more capable of performing ADL. Moreover, interviewees emphasized that when someone is less able to perform ADL independently, this complicates the observation of alterations in ADL.

Speech therapist M.: “Symptoms like not understanding how to perform a task, how to brush your teeth (…), how to dress up, those are not indicative for dementia in people with SPI(M)D, because in general we do that for them.”

#### Behavioral and Psychological Changes

All interviewees highlighted that since the onset of (suspected) dementia they had observed behavioral and psychological changes. Particularly, an increase in anxious behavior as compared to the pre-existing life-long characteristic behavior was noticed.

Psychologist C.: “Observable behavior related to dementia is anxiety and nervousness, for instance, an anxious facial expression, (…) or hesitant to walk. Screaming, that can of course also be a sign of anxiety.”

Moreover, since the onset of (suspected) dementia interviewees had frequently observed an increase in apathetic behavior, sleeping problems, irritability, obstinate behavior, restlessness/stereotypic behavior and a decrease in eating/drinking behavior. Interviewees emphasized that often a combination of such changes was observed in specific situations.

Speech therapist M.: “Signs which are often observed during eating and drinking are restlessness, crying or falling asleep at the table. (…) A person could also be less alert.”

Moreover, it was emphasized that it is difficult to observe psychotic and depressive behavior in people with SPI(M)D.

Psychologist C.: “Unhappiness can be observed when the entire appearance of a person changes, for instance, hollow eyes or keeping your head down. It remains very complex (…). It could be dementia, but it could also be a depression.”

#### Motor Changes

A deterioration of walking skills accompanied by increased balance problems and wheelchair use were frequently observed by interviewees.

Physiotherapist P.: “Persons with walking skills at baseline, lose at a certain moment their ability to walk and eventually become dependent on a wheelchair. However, then it is often already obvious that those individuals have dementia.”

Furthermore, interviewees stated that chewing and swallowing became progressively more difficult with the onset of (suspected) dementia.

Physician J.: “People with SPI(M)D often already have swallowing problems, but it becomes progressively worse (…). I think that is a sign.”

#### Medical Comorbidities

Interviewees stated that they had observed medical comorbidities like the onset of epilepsy, becoming incontinent and weight changes with the onset of (suspected) dementia.

Nurse specialist S.: “Particularly in people with Down syndrome, epilepsy is something that can be associated with dementia.”

## Discussion

Using a survey and semi-structured interviews, an inventory of practice-based observations of dementia symptoms in people with SPI(M)D was obtained. Survey data indicated that the most frequently observed symptom concerned a decline in ADL functioning, followed by behavioral and psychological symptoms of dementia, in particular changes in irritable, eating/drinking, anxious and apathetic behavior. To lesser extent cognitive symptoms, motor changes and medical comorbidities were observed. Subsequently, interviews provided a richer and more in-depth perspective on symptoms covered in the survey. Cognitive symptoms were generally observed when persons had verbal communication or walking skills at baseline, whereas behavioral and psychological changes were mostly noticed regardless of having such baseline skills. Moreover, motor changes were particularly observed when persons were at baseline able to walk. Lastly, changes in ADL functioning and medical comorbidities were observed in people with and people without walking skills at baseline.

To timely recognize and diagnose dementia in SPI(M)D insights in the symptomology are needed. This also contributes to better understanding and making informed choices (Dekker, Wissing et al., 2021). Recently, we conducted a systematic literature review to identify observable symptoms in the scarce literature about dementia in this population (Wissing et al., 2021). Given the very limited number of studies, we conducted an explorative focus group study to obtain practice-based experiences (Dekker, Wissing et al., 2021). This study was the next step, to further identify practice-based observations of dementia symptoms in this population. Hereafter, we contextualize survey and interview results with the outcomes of the systematic literature review and focus groups (Table 4; Dekker, Wissing et al., 2021; Wissing et al., 2021).

**Table 4.** Comparison of dementia symptoms in people with SPI(M)D obtained with different research methods.

	Symptoms	Survey	Interviews	Focus groups^1^	Literature review^2^
Cognitive changes	↓ Memory	✔	✔	✔	✔
	↓ Orientation in place	✔	✔	✔	✔
	↓ Language skills	✔	✔	✔	✔
	↓ Responsiveness	✔	✔	✔	
	↓ Person recognition	✔	✔	✔	
	↓ Awareness of proper order	✔	✔	✔	
	↓ Object recognition	✔	✔	✔	
	↓ Orientation in time	✔	✔	✔	
	↓ Preference for (favorite) objects	✔	✔	✔	
	↓ Understanding visual images/spatial relationships	✔	✔	✔	
	↓ Concentration	✔	✔		
	↑ Losing objects	✔	✔		
	↑ Sensory sensitivities	✔	✔		
	↓ Planning	✔			
	↓ Problem solving	✔			
	↓ Judgment	✔			
	↓ Sound recognition		✔		
ADL	↓ ADL	✔	✔	✔*	✔
Behavioral and psychological changes	↑ Irritable behavior	✔	✔	✔	✔
	↓ Eating/drinking behavior	✔	✔	✔	✔
	↑ Apathetic behavior	✔	✔	✔	✔
	↑ Sleeping problems	✔	✔	✔	✔
	↑ Restless/stereotypic behavior	✔	✔	✔	✔
	↑ Aggressive behavior	✔	✔	✔	✔
	↑ Anxious behavior	✔	✔	✔	
	↑ Obstinate behavior	✔	✔	✔	
	↑ Disinhibited behavior	✔		✔	✔
	↑ Depressive behavior	✔	✔	✔	
	↑ Psychotic behavior	✔	✔	✔	
Motor changes	↓ Walking	✔	✔	✔	✔
	↑ Wheelchair use	✔	✔	✔	
	↓ Balance	✔	✔	✔	
	↑ Fall frequency	✔	✔	✔	
	↑ Swallowing problems	✔	✔	✔	
	↑ Stiffness	✔	✔	✔	
	↑ Cramps	✔	✔	✔	
	↓ Body awareness	✔	✔	✔	
	↓ Muscle strength	✔	✔	✔	
	↓ Motor skills	✔	✔	✔	
	↓ Movement speed	✔	✔		
	↑ Fetal sitting/laying position	✔	✔		
	↑ Tremor	✔			
Medical comorbidities	↓ Weight	✔	✔	✔	✔
	↑ Incontinence	✔	✔	✔	✔
	↑ Epilepsy	✔	✔	✔	✔
	↑ Bedridden	✔	✔		✔
	↑ Pain	✔		✔	
	↓ Taste sensation	✔		✔	
	↓ Bowel movements		✔		

This table provides a comparison of dementia symptoms reported in the survey, interviews with previously published findings using two other research methods, namely focus groups (Dekker, Wissing et al., 2021) and systematic literature review (Wissing et al., 2021). Symptoms are categorized in five symptom domains, which is in line with dementia criteria (American Psychiatric Association, 2013; McKhann et al., 2011; World Health Organization, 2018) and literature (Dekker, Ulgiati et al., 2021; Ries, 2018; Strydom et al., 2010). ✔ indicates that a symptom was reported in a research method. For behavioral and psychological changes, motor changes and medical comorbidities only the most prominently reported symptoms are presented. Legend: ↓ = decrease compared to baseline level of functioning, ↑ = increase compared to baseline level of functioning. Baseline level of functioning is the highest level of functioning before dementia-related decline occurred. *Symptoms reported in focus groups were categorized based on the daily contexts in which they were often observed in practice. Therefore, a decline in activities of daily living (ADL) functioning was addressed in various contexts and symptoms.

### Cognitive Changes

An ID is characterized by deficits in cognitive functioning, e.g., deficits in reasoning, problem solving, planning, judgment (American Psychiatric Association, 2013). If cognitive skills have not or hardly been developed at baseline, such skills cannot decline and therefore cannot be indicative of dementia (Llewellyn, 2011; Sheehan et al., 2015). Consequently, one could hypothesize that cognitive decline would be less observable in people with SPI(M)D. However, results from the survey, interviews as well as previous findings in focus groups (Dekker, Wissing et al., 2021) and literature (Wissing et al., 2021) jointly show that, despite the low baseline level of functioning, cognitive alterations like memory loss, disorientation in place and deterioration in language skills are observable in this population (Table 4). It should be noted that interviewees emphasized that such changes are (more easily) observed when individuals have verbal communication or walking skills at baseline. As expected, higher cognitive functions such as planning, problem solving and judgment were not mentioned in interviews, focus groups (Dekker, Wissing et al., 2021) and literature (Wissing et al., 2021), and hardly addressed in the survey (Table 4).

### ADL Changes

In addition to cognitive decline, another prominent sign of dementia concerns decline in ADL functioning(Alzheimer’s Association, 2021; American Psychiatric Association, 2013; McKhann et al., 2011; World Health Organization, 2018). In people with SPI(M)D and (suspected) dementia this was prominently observed by survey respondents and interviewees. These findings underline previous findings in focus groups (Dekker, Wissing etal., 2021) and literature (Wissing etal., 2021); Table 4). However, the way in which decline in ADL manifests depends on someone’s baseline functioning, as already addressed by Benejam (2009). Interviews showed that in most persons a decline in eating/drinking skills was observed, whereas a decline in dressing, toilet use and stair climbing was only observable in individuals more capable of performing ADL.

### Behavioral and Psychological Changes

Behavioral and psychological symptoms of dementia can be observed in all types of dementia and are most observable for caregivers (Engelborghs et al., 2005; Finkel, 2000). Indeed, results from the survey, interviews as well as previous findings of focus groups (Dekker, Wissing et al., 2021) and literature (Wissing et al., 2021) jointly showed that behavioral changes such as increased irritable, restless/stereotypic, aggressive, apathetic behavior and decreased eating/drinking behavior can be observed in persons with SPI(M)D and (suspected) dementia (Table 4). In line with recent findings in two large studies on dementia in people with Down syndrome with mild, moderate and severe ID (Dekker, Ulgiati et al., 2021; Dekker et al., 2018), prominent behavioral and psychological symptoms of dementia were changes in irritable, eating/drinking, anxious, apathetic, restless/ stereotypic behavior and sleeping problems, whereas psychotic behavior was less frequently observed. Communication of individuals with SPI(M)D is less verbal, making it complex to accurately elucidate the inner experience of delusions and hallucinations (Cooper & Smiley, 2007). In contrast to Dekker et al. (2018, 2021), changes in depressive behavior were less frequently reported in individuals with SPI(M)D and (suspected) dementia. Recognizing and differentiating between depression and depressive symptoms related to dementia is particularly difficult in this population because persons with limited verbal communication skills cannot report their mood and do not have the cognitive level for specific symptoms that classically characterize depression, such as doom mongering or being tired of life (Dekker et al., 2015, 2018; K. M. Evans et al., 1999).

### Motor Changes

In the general population, motor changes such as gait changes and diminished postural control (balance and falls) are observed in individuals with dementia (Ries, 2018). Survey and interview results demonstrated that in people with SPI(M)D and (suspected) dementia such motor changes were also observed in those with walking skills at baseline. Moreover, both research methods showed that since the onset of (suspected) dementia swallowing problems increased in this population. These motor changes were also found in the focus group study (Dekker, Wissing et al., 2021); Table 4).

### Medical Comorbidities

Incontinence, onset of epilepsy and weight changes are recognized as medical comorbidities with dementia in the general population (Kurrle et al., 2012) and people with Down syndrome (Strydom et al., 2010). Similarly, these comorbidities were reported in survey and interviews and are also consistent with the findings of the focus groups (Dekker, Wissing et al., 2021) and literature (Wissing et al., 2021). With respect to epilepsy, interviewees stressed that the onset of epilepsy was particularly observed in those with Down syndrome, which is in line with results of other studies focusing on late onset myoclonic epilepsy in Down syndrome (Altuna et al., 2021; Aller-Alvarez et al., 2017; Menéndez, 2005).

### Study Strengths

One of the strengths of this study is the mixed methods design comprising a quantitative survey and qualitative interviews to identify practice-based observations of dementia symptoms in people with SPI(M)D. To the best of our knowledge, this study is the first to examine whether dementia symptoms described in literature are observed in daily practice in this population. Furthermore, a richer and more in-depth perspective on symptoms covered in the survey was obtained by conducting interviews with care professionals. A strength of the interviews is the purposive sampling of care professionals having vast experience in signaling/diagnosing dementia in this population. Furthermore, the heterogeneity of this population was considered through inductive content analysis of transcripts, enabling us to refine results (categorization of symptoms) in relation verbal communication and walking skills at baseline.

### Study Limitations

A first potential limitation concerns the fact that a rather small number of family members completed the survey. This might be related to the fact that most elderly with SPI(M)D have spent much of their lives in a care institution (Johnson & Traustadóttir, 2005), which could have an impact on the (more distant) family involvement. Secondly, providing care is key priority when working with people with SPI(M)D, and therefore care professionals might not always had time to complete the survey. Thirdly, care professionals provide care to people with different levels of functioning, and therefore could have referred to some signs of dementia in people with mild/moderate ID. In the survey (introductory texts) and interviews, the focus on SPI(M)D was clearly emphasized. Fourthly, given the complexity of diagnosing dementia is this population a diagnosis is often not formally established. Therefore, family members and care professionals could have referred to symptoms caused by others conditions that mimic dementia. This is the result of limited knowledge about dementia symptoms in people with SPI(M)D. It underlines the relevance of research on the symptomatology of dementia in this population.

### Future Implications

Diagnosing dementia in people with SPI(M)D is an often long and complex process. It is already quite difficult to establish a general diagnosis of dementia, let alone that a diagnosis of the subtype of dementia (e.g., AD, dementia with Lewy bodies, vascular dementia or frontotemporal dementia) is established (Burt et al., 1998; Day, 1985; Duggan et al., 1996; Margallo-Lana et al., 2007; Reid & Aungle, 1974). Diagnosing dementia requires a proper diagnostic procedure. Dementia-like symptoms could be caused by – often treatable – conditions, also referred to as pseudo-dementias (Zigman, 2013; Zigman et al., 2008). Therefore, potential other causes, such as depression, delirium, vision or hearing problems, hypothyroidism, medication use, sleep apnea or vitamin B12 deficiency – should be ruled out as much as possible before establishing a diagnosis of dementia (Moriconi et al., 2015; Scott & Barrett, 2007). Moreover, tests could be used to monitor the progression of reduction in functioning over time. However, currently there are hardly any validated direct neuropsychological tests and informant-based dementia questionnairesavailable to (early) diagnose dementia in people with SPI(M)D (Elliott-King et al., 2016; Esbensen et al., 2017; Fletcher et al., 2016; Hon et al., 1999; Keller et al., 2016; McKenzie et al., 2018). An inventory of observable dementia symptoms through a survey and interviews, together with findings from literature (Wissing et al., 2021) and focus groups (Dekker, Wissing et al., 2021) provide a first essential step for developing a dedicated dementia screening instrument for people with SPI(M)D. The development process may be aided by identifying relevant items within existing dementia screening instruments primarily applicable to people with mild and moderate ID.

### Conclusions

This study provided an overview of observable dementia symptoms in people with SPI(M)D. Particularly, a decline in ADL functioning and behavioral and psychological symptoms like increased irritable, anxious, apathetic behavior and decreased eating/drinking behavior were recognized. To a lesser extent cognitive symptoms like memory loss, disorientation in place and deterioration in language skills were observed, particularly in those with verbal communication or walking skills at baseline. Furthermore, motor changes and medical comorbidities were reported. The inventory of symptoms in this study together with findings from literature (Wissing et al., 2021) and focus groups (Dekker, Wissing et al., 2021) pave the way for developing a dedicated screening instrument for dementia in people with SPI(M)D.
